# Construction of Vertical 2D Open Hierarchical NiCoS_x_ Nanosheet Arrays for High-Performance Alkaline Zinc Batteries

**DOI:** 10.3390/nano16120766

**Published:** 2026-06-18

**Authors:** Junqing Huang, Xiaodong Liang, Qian Zhang, Luyang Ge, Jiangtao Pan, Debing Long, Xiyan Bao, Xiaolin Wu, Houzhao Wan

**Affiliations:** 1 School of Physics and Electronic-Information Engineering, Hubei Engineering University, Xiaogan 432000, China; 202321119012920@stu.hubu.edu.cn (J.H.); 202511119010210@stu.hubu.edu.cn (X.L.); debinglong@foxmail.com (D.L.); 2 Hubei Key Laboratory of Micro-Nanoelectronic Materials and Devices, School of Integrated Circuits, Hubei University, Wuhan 430062, China; qianzzz@stu.hubu.edu.cn (Q.Z.); luyangg@stu.hubu.edu.cn (L.G.); 202321119012798@stu.hubu.edu.cn (J.P.); 202321119012825@stu.hubu.edu.cn (X.B.)

**Keywords:** alkaline zinc battery, cobalt sulfide, in situ sulfidation, two-dimensional nanosheets, electrode material

## Abstract

Alkaline nickel zinc batteries feature high safety, low cost and eco-friendly characteristics, making them highly promising for large-scale energy storage deployment. However, their practical application is severely constrained by the cathode’s electrical conductivity, available active sites, and cycling stability. Herein, vertical 2D hierarchical flake-like NiCoS_x_ arrays were in situ grown on nickel foam (NF) via a facile alkali-free solvothermal and in situ sulfidation approach. This highly interconnected and open porous flaky structure significantly shortens the ion diffusion pathways, exposes abundant redox-active sites, and accelerates electron transport, imparting excellent rate performance and superior long-cycle stability to the material. The optimized NiCoS_x_/NF electrode achieves a high specific capacity of 323 mAh g^−1^ at 0.5 A g^−1^, along with excellent capacity retention capability. Assembled with a commercial Zn anode, the NiCoS_x_/NF//Zn full battery delivers 124 mAh g^−1^ at 3 A g^−1^, and maintains 112.5% of the initial capacity after 500 cyclic tests. Moreover, the assembled NiCoS_x_/NF//Zn full cell possesses a high energy density of 615.2 Wh kg^−1^ along with a power density of 38.6 kW kg^−1^ (based on the mass of positive electrode active materials). This unique vertical 2D open hierarchical structure plays a crucial role in enhancing the electrochemical performance of cobalt sulfide cathodes and provides valuable insights for the design of high-performance alkaline zinc-based battery electrodes.

## 1. Introduction

In the current development of battery technology, the electrode materials field faces numerous challenges that significantly limit the application prospects of zinc batteries (NZBs) [1,2]. Among these, the dendrite issue in zinc anodes is particularly thorny. Its frequent occurrence not only reduces the Coulombic efficiency of batteries but may also penetrate the separator, triggering safety hazards such as short circuits and causing a sharp decline in battery capacity [3,4]. From the perspective of the overall architecture of alkaline NZB systems, the self-dissolution of cathode materials has become a key obstacle restricting the improvement of their energy density [5,6]. Cobalt sulfides stand out as advanced electrode candidates by virtue of superior electrochemical activity, favorable electronic conductivity, and outstanding mechanical and thermal stability, showing better performance than corresponding oxides and hydroxides [7]. Benefiting from the synergy between the multi-valent elements nickel and cobalt, they offer more abundant redox active sites than single-component sulfide materials, which can greatly enhance electrochemical performance and make them well-suited for application in alkaline electrolytes [8,9].

Mixed-valence transition metal systems are highly promising for electrochemical energy storage because of their tunable valence states, rich redox activity, and improved electronic conductivity [10]. The synergistic electronic interactions between mixed-valence metal sites can effectively enhance electron transport, structural stability, and reversible redox kinetics, which are critical for advanced alkaline zinc batteries (summarized in Table S1) [11].

Among the numerous approaches for preparing high-performance cobalt sulfides, existing studies have amply demonstrated that in situ conversion using corresponding oxide or hydroxide precursors is a highly effective strategy [12]. Throughout the transformation procedure, the precursor inherits its initial structure and morphology perfectly. The obtained cobalt sulfides deliver greatly improved electrochemical properties and superior cycling durability, which offers a feasible route for scalable fabrication of high-performance cobalt sulfide electrodes . For instance, Zha et al. designed and constructed a cobalt sulfide electrode with a highly open structure, which demonstrated exceptional supercapacitive performance and excellent cycling stability [13]. Yan et al. employed a two-step method to in situ synthesize on nickel foam (NF) a uniform array of bimetallic NiCoS nanorods assembled from vertically oriented nanosheets, greatly increasing the number of exposed active sites and achieving faster charge-transfer rates as well as a lower Tafel slope [14]. Dai and coworkers deposited NiCo-LDH nanosheets in situ on NiCoS nanotube arrays, constructing a distinctive 3D core shell heterostructure. This unique architecture not only enhances the material’s electronic conductivity but also offers ample active sites and efficient ion/electron transfer pathways. The NiCoS@NiCo-LDH electrode achieves a high capacity of 312 mAh g^−1^ (0.624 mAh cm^−2^) at 2 mA cm^−2^, retaining 90% of its initial capacity even when the current density is increased to 10 mA cm^−2^. Building upon this work [15], Qian et al. utilized a simple anion-exchange reaction to control different sulfurization concentrations, thereby fabricating vertically oriented NiCo_2_S_x_ nanoneedle arrays on nickel foam. Among these samples, the optimal one NiCo_2_S_x_-600 displayed a uniform and complete hollow nanoneedle morphology, offering rapid ion-transport pathways and enabling effective modulation of the electronic structure through Co^3+^. The as-fabricated NiCo_2_S_x_@NF electrode achieves an excellent specific capacity of 121.9 mAh g^−1^ at 3 A g^−1^, and maintains a high capacity retention of 91.1% after 500 consecutive cycles. Furthermore, the alkaline NiCo_2_S_x_//Zn battery assembled with this electrode reaches a prominent energy density of 636.3 Wh kg^−1^ and a power density of 40.9 kW kg^−1^ [16].

However, conventional NiCoS_x_ electrodes are plagued by intrinsic limitations, including prolonged ion diffusion pathways, inadequate exposure of active sites, and proneness to structural pulverization and collapse during charge discharge cycling, which severely limit further improvements in their rate performance and cycle life [17,18]. In contrast, two-dimensional lamellar nanoarchitectures display exceptional merits, including a large specific surface area, fully exposed highly active crystal facets, shorter ion and electron transport distances, and enhanced structural stability and flexibility [19,20]. These features can fundamentally accelerate electrode reaction kinetics and improve structural durability, making them an ideal structural form for overcoming current performance bottlenecks [21,22]. Unfortunately, to date, there still lacks a mature method for the controlled synthesis of uniform, vertically aligned, ultrathin two-dimensional flaky NiCoS_x_ on nickel foam substrates, and the structure-property relationships between the two-dimensional flaky architecture and electrochemical performance remain poorly understood and require more systematic and in-depth research [23,24].

In this study, we employed an alkali-free “solvo-thermal and in situ hydrolysis” strategy. By carefully controlling the cobalt ratio and solvo-thermal conditions, we successfully prepared in situ, flaky NiCoS_x_/NF electrodes. This highly interconnected and open porous sheet-like architecture effectively shortens ion diffusion paths, exposes abundant redox-active sites, and boosts electron transport efficiency, thus endowing the material with exceptional rate capability and long-cycle stability. The optimized NiCoS_x_/NF electrode exhibits a specific capacity of 323 mAh g^−1^ at 0.5 A g^−1^ and retains an outstanding capacity retention of 196.8% after 1000 cycles at 5 A g^−1^. The superior performance of this flaky morphology-enhancing conductivity, increasing active-site exposure, enhances rate performance and reinforces cycling stability, offering both experimental and theoretical guidance for designing high-performance cathode materials.

## 2. Experiment

### 2.1. Chemical Reagents and Reagents

All other analytical grade reagents were used as purchased without further purification. Nickel acetate tetrahydrate (C_4_H_6_NiO_4_·4H_2_O), cobalt acetate tetrahydrate (C_4_H_4_CoO_4_·4H_2_O), and sodium sulfide nonahydrate (Na_2_S·9H_2_O, 98%) were acquired from Aladdin Industrial Co., Ltd. (Shanghai, China). Anhydrous ethanol was supplied by Sinopharm Chemical Reagent Co., Ltd. (Shanghai, China). All aqueous solutions were prepared with deionized water (DW).

### 2.2. Preparation Method for NiCo/NF Electrode

Preparation of the NiCo/NF precursor: The nickel foam was immersed in 0.1 M hydrochloric acid solution and sonicated for 30 min to eliminate the surface oxide layer. Subsequently, it was rinsed repeatedly with deionized water and ethanol, followed by vacuum drying for 12 h. Dissolve 0.6 mmol of nickel acetate tetrahydrate (C_4_H_6_NiO_4_∙4H_2_O) and 0.6 mmol of cobalt acetate tetrahydrate (C_4_H_6_CoO_4_∙4H_2_O) completely in 60 mL of methanol solution by stirring. The resulting solution was transferred into a 100 mL stainless steel autoclave lined with polytetrafluoroethylene (PTFE). Subsequently, the pre-treated nickel foam was placed into the autoclave liner and sonicated for 30 min to guarantee thorough wetting. Then, place the autoclave in an oven set at 160 °C for 12 h. After cooling to room temperature, the nickel foam was taken out and washed repeatedly with deionized water and ethanol, and dired in a vacuum oven. The result is an array of NiCo/NF precursor nanosheets supported and adhered onto the nickel foam. Repeat the above operation according to cobalt ratios of 3:1, 2:1, 1:2, and 1:3 to obtain NiCo/NF precursor nanosheet arrays with different cobalt ratios.

Preparation of NiCoSx/NF: The as-prepared NiCo/NF was placed into a 100 mL Teflon-lined stainless steel autoclave filled with 60 mL of 0.12 M Na_2_S aqueous solution, and kept at 120 °C for 6 h. After the reaction, the black product was washed thoroughly with deionized water and anhydrous ethanol, followed by vacuum drying for 24 h to obtain the final NiCoS_x_/NF product. Repeat the above operations according to the different cobalt ratios mentioned earlier, and name them Ni_3_CoS_x_/NF, Ni_2_CoS_x_/NF, NiCo_2_S_x_/NF, and NiCo_3_S_x_/NF, respectively.

### 2.3. Characterization Methods

X-ray diffraction (XRD) was utilized to identify the phase components and crystalline structure of the as-prepared samples. In the present study, XRD characterizations were carried out on a Bruker (Bruker AXS SE, Karlsruhe, Germany) D8A25 diffractometer operated at a maximum power of 3 kW with Cu Kα radiation (λ = 1.5406 Å). All data were recorded in a 2θ range from 10° to 90° at a scanning step of 0.0001°. SEM (JSM-7100F, JEOL (Tokyo, Japan)) and TEM were employed to investigate the microstructures and morphologies. EDX was used to examine the elemental distribution. FTIR spectra were recorded on a Thermo Fisher Scientific (Wilmington, DE, USA) spectrometer to characterize the chemical bonding features.

### 2.4. Electrochemical Performance Testing Methods

The mass loading of active materials in the as-prepared electrodes was about 1–2 mg cm^−2^. The electrochemical properties of the individual electrode were assessed in a three-electrode configuration using a CHI760E electrochemical workstation (Chenhua, Shanghai, China). The active material was employed as the working electrode, while a Hg/HgO electrode and platinum foil acted as the reference and counter electrodes, respectively. A 6 M KOH solution was utilized as the electrolyte. The specific capacity of the electrode was evaluated by galvanostatic charge–discharge (GCD) tests. The mass-specific capacitance (C, mAh g^−1^) at different current densities was calculated based on the following equation to estimate the charge-storage performance.

(1) $$C_{m}=\frac{I\cdot \Delta t}{m}$$

The mass-specific capacity (C_m_) is expressed in mAh g^−1^, where I represents the applied current, m denotes the mass of the active material, and Δt stands for the discharge time. The gravimetric energy density (E, Wh kg^−1^) and power density (P, kW kg^−1^) of the full battery were calculated using the following equations:

(2) $$E=\frac{C\times V_{avg}}{m}$$

(3) $$P=\frac{E}{t}$$

where C refers to the discharge specific capacity (Ah g^−1^), V_av9_ represents the average discharge voltage (V), I is the discharge current (A), m is the mass of cathode active material (g), and t denotes the discharge time (h).

The electrochemical behaviors of aqueous alkaline Ni-Zn batteries were systematically evaluated using 4 M KOH solution and saturated zinc acetate aqueous solution as the electrolytes. The as-fabricated electrode materials were cut into circular wafers with an area of 1.13 cm^2^ and assembled into the positive shell of coin cells. An appropriate amount of electrolyte was dropped via a pipette to fully infiltrate the electrode wafers, followed by the placement of a hydrophilic separator with additional electrolyte supplementation. Subsequently, the zinc anode was installed, and the coin cells were sealed using a crimping machine. A two-electrode configuration was fabricated using NiCoS_x_/NF as the cathode and commercial zinc foil as the anode. Cyclic voltammetry (CV), galvanostatic charge–discharge (GCD), and electrochemical impedance spectroscopy (EIS) tests were performed. The rate performance and cycling durability of the batteries were further assessed via a Neware (Shenzhen, China) battery testing system.

## 3. Results and Discussion

### 3.1. Synthesis and Characterization of NiCo/NF

**Figure 1:**
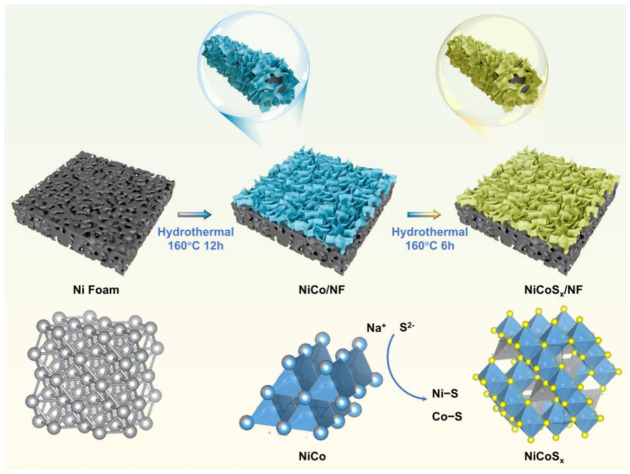
Schematic of NiCoS_x_/NF nanosheets: synthesis mechanism.

This work employs a two-step controllable synthesis strategy combining in situ hydrothermal growth with anionic sulfidation exchange to precisely fabricate vertically grown, uniform 2D NiCoS_x_ nanosheet arrays on a 3D porous conductive nickel foam (NF) substrate . First, commercial nickel foam is subjected to ultrasonic pretreatment in dilute hydrochloric acid, which efficiently etches away the naturally formed oxide passivation layer and roughens the microsurface, significantly enhancing the substrate’s hydrophilicity and exposing more metallic active sites [25]. This creates excellent nucleation sites and strong interfacial adhesion for the subsequent uniform in situ growth of active materials. In the first step, during the methanol-based hydrothermal reaction, Ni^2+^ and Co^2+^ dissolved in methanol undergo slow co-precipitation via hydrolysis under a sealed environment at 160 °C. The low polarity and low surface tension properties of methanol effectively regulate the anisotropic crystal growth, inducing the cobalt precursors to self-assemble along preferred two-dimensional orientations, thereby forming a vertically interlaced array of ultrathin nanosheets that are firmly anchored onto the three-dimensional interconnected nickel foam skeleton [26]. By precisely controlling the Ni/Co molar ratio (3:1, 2:1, 1:2, or 1:3), we can directionally optimize the thickness of the precursor nanosheets, the interlayer spacing, and the openness of the array, enabling precise control over the microstructure and electronic properties [27]. The as-fabricated NiCo/NF precursor possesses a hierarchical 2D layered structure, large specific surface area, and excellent intrinsic electrical conductivity. In the second step, a solvothermal sulfidation process uses NiCo/NF as a self-sacrificing template, with Na_2_S providing S^2−^ ions. Under hydrothermal conditions at 120 °C, S^2−^ ions diffuse inward from the precursor surface through ion diffusion and undergo an in situ anionic exchange reaction with Ni and Co lattice sites. Oxygen-containing functional groups and hydroxyl groups are replaced by S^2−^ ions, while the Ni-Co metal coordination framework retains its original two-dimensional layered morphology and is transformed in situ into a bimetallic sulfide NiCoS_x._ This process, guided by the template and driven by anionic topological transformation, fully preserves the precursor’s two-dimensional array structure, preventing particle agglomeration and structural collapse, and maintaining a highly open multi-level pore network. The in situ sulfidation ensures uniform formation of Ni-S and Co-S chemical bonds, optimizes the electronic coordination environment around metal sites, and enhances the synergistic electron transfer effect between the two metals. The three-dimensional nickel foam and the active nanosheet array form a tightly integrated, self-supporting structure without the need for additional binders, greatly reducing interfacial contact resistance and providing a continuous conductive network for fast ion and electron transport [28]. As illustrated in Figure 1, the constructed NiCoS_x_/NF array inherits a vertically oriented sheet-like morphology, which significantly enhances the intrinsic conductivity of the bimetallic sulfide. The rich interlayer spacing and hierarchical porosity effectively alleviate volume variation during cycling, thus enhancing the long-term cyclic stability of the electrode. The ultra-large electrochemically active surface area exposes abundant redox sites, facilitates ion transport and reaction kinetics, and thus endows the material with excellent rate capability and high specific capacity [29]. Furthermore, regulating the initial Ni/Co ratio can further optimize the phase composition, defect concentration, and modulate the electronic band structure of the sulfide, fully unlocking its intrinsic electrochemical activity, together with outstanding rate performance and high specific capacity. By tuning the initial Ni/Co ratio, further optimization can be achieved.

**Figure 2:**
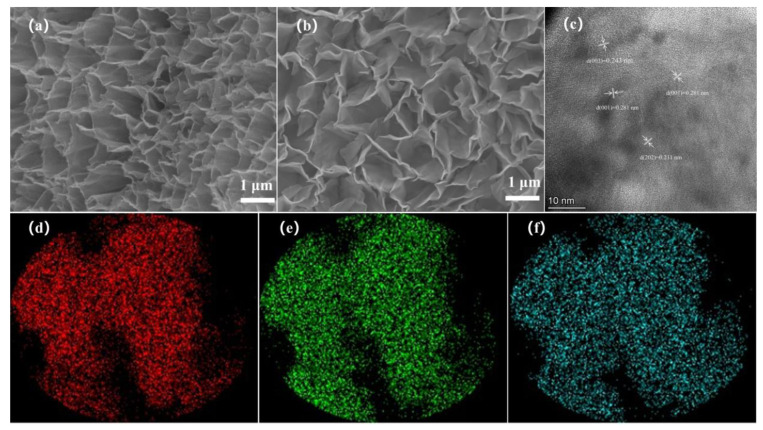
(**a**) NiCo/NF precursor (SEM); (**b**) NiCoS_x_/NF (SEM); (**c**) NiCoS_x_/NF (TEM); (**d**–**f**) EDS mappings of Ni, Co, S in NiCoS_x_/NF.

Figure 2a displays the SEM morphology of the hydrothermally synthesized NiCo/NF (Figure S1a). At a magnification of 15,000×, it can be clearly observed that the initially bare substrate is uniformly covered with a large number of highly interconnected NiCo nanosheets, providing a favorable foundation for the subsequent formation of stable sulfide nanosheet structures [30]. Under different cobalt ratios, after the sulfuration process, a nanosheet structure also appears, but the nanosheet structure is not complete (Figure S1b–f). When NiCo/NF is sulfurized to form NiCoS_x_/NF (Figure 2b), the structural integrity of the electrode is maximally preserved, still maintaining the highly open, interconnected nanosheet morphology of the precursor. This phenomenon indicates that the sulfurization process occurs in situ without destroying the original sheet-like structure, and this stable sheet-like architecture is key to the excellent electrode performance. Figure 2c presents the TEM image of NiCoS_x_/NF, where the lattice fringe spacings show no significant changes, with values of d(110) = 0.281 nm, d(202) = 0.211 nm, and d(033) = 0.243 nm. The regular and stable crystal lattice structure serves as the microscopic basis for the overall stability of the nanosheet architecture, further maintaining the structural integrity of the electrode during cycling. The EDS elemental mapping images (Figure 2d–f and Figure S2a) clearly show a uniform distribution of Ni, Co, and S elements throughout the sample. This uniform distribution ensures the homogeneity and stability of the nanosheet structure and strongly confirms the successful preparation of the NiCoS_x_/NF material. In summary, the precursor exhibits a uniform, continuous, and highly interconnected 2D sheet-like structure, with sheets growing vertically on the nickel foam surface and forming open channels between them [31]. After sulfurization, the sheet-like morphology is completely retained without noticeable fragmentation, agglomeration, or structural collapse; only the sheet surfaces become rougher, forming a hierarchical porous structure. Adjusting the cobalt ratio significantly affects the integrity of the sheets; only at an optimal ratio can a stable and complete nanosheet structure be formed. This stable structure is the core prerequisite for guaranteeing electrode performance.

**Figure 3:**
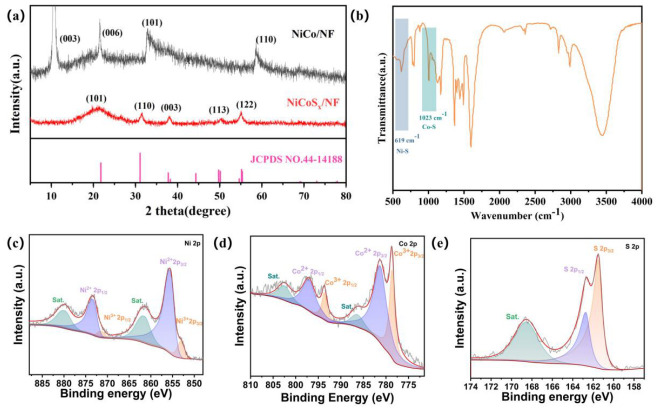
(**a**) XRD patterns of the NiCo/NF precursor and NiCoS_x_/NF; (**b**) FTIR spectrum of NiCoS_x_/NF; (**c**–**e**) Ni 2p, Co 2p and S 2p high-resolution XPS spectra of NiCoS_x_/NF.

XRD characterization was performed to verify the crystal structure and phase purity of the samples, as shown in Figure 3a. The characterization results of the NiCo/NF precursor indicate that a hydrotalcite-like NiCo-LDH was successfully formed. After sulfidation, the characteristic peaks of NiCo-LDH almost disappeared. In the NiCoSx/NF spectrum, distinct characteristic diffraction peaks emerge at 21.7°, 30.9°, 37.8°, 49.7° and 55.1°, corresponding respectively to the (101), (110), (003), (113) and (122) planes of hexagonal cobalt sulfide (JCPDS No. 44-1418). Moreover, no impurity signals were detected, confirming that the product obtained by controlling the cobalt ratio exhibits high phase purity [32]. A highly phase-pure crystal structure is crucial for forming a stable nanosheet-like structure, which further ensures the structural stability and electrochemical performance of the electrode. To further clarify the composition and structure of the material, FTIR characterization was conducted, with the results presented in Figure 3b. In the FTIR spectrum, two prominent peaks at 619 cm^−1^ and 1023 cm^−1^ correspond to the characteristic vibrational modes of Ni-S and Co-S bonds, respectively, providing strong evidence for the successful synthesis of the sulfided product NiCoS_x_ [33]. The stable chemical bonding is an essential foundation for maintaining the structural stability of the nanosheet-like morphology [34]. XPS analysis was conducted on the NiCoSx/NF sample to determine the valence states of each element (Figure S2b). Deconvolution via Gaussian fitting indicates that both Ni 2p and S 2p profiles present two clear spin orbit split doublets and two satellite signals. The characteristic peaks located at 873.2 eV and 855.3 eV are assigned to Ni 2p_1/2_ and Ni 2p_3/2_ in Figure 3c. In Figure 3e, the binding energies at 162.6 eV and 161.2 eV are assigned to S 2p_1/2_ and S 2p_3/2_, respectively. In Figure 3d, the binding energies of Co 2p_3/2_ at 778.3 eV and Co 2p_1/2_ at 793.2 eV are characteristic of the spin orbit features of Co^3+^, while those at 780.3 eV for Co 2p_3/2_ and 797.3 eV for Co 2p_1/2_ correspond to Co^2+^, confirming the coexistence of Co^2+^ and Co^3+^. Notably, Co^3+^ in NiCoS_x_/NF exhibits a high binding energy state, which endows the material with superior stability, effectively optimizing its electronic conductivity and structural integrity [35]. Collectively, the XPS results unequivocally confirm the successful synthesis of a cobalt bimetallic sulfide with coexisting multiple valence states, where the electronic synergy between the two metals effectively optimizes the electronic coordination environment of the material.

### 3.2. Electrochemical Performance Testing and Analysis

**Figure 4:**
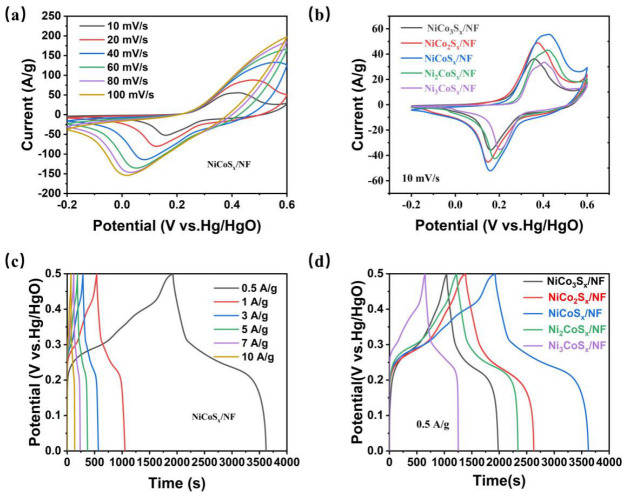
(**a**) CV curves of NiCoS_x_/NF electrode; (**b**) CV comparison of electrodes with different Ni/Co ratios at 10 mV s^−1^; (**c**) GCD profiles of NiCoS_x_/NF electrode; (**d**) GCD curves of various Ni/Co ratio electrodes at 0.5 A g^−1^.

The tested electrode sheets were punched into circular discs with a fixed diameter (effective area ≈ 1.13 cm^2^), and the loading mass of active materials was controlled at 1–2 mg. The obtained discs were then fixed as working electrodes. Electrochemical measurements including CV, GCD and EIS were carried out for samples with different Ni/Co molar ratios in 6 M KOH electrolyte, using Hg/HgO as the reference electrode and platinum sheet as the counter electrode (Figures S3a–f and S4a–f). As displayed in Figure 4a, CV curves were recorded within a potential window of −0.2 V to 0.6 V at scan rates ranging from 10 to 100 mV s^−1^. The CV curves of the NiCoS_x_/NF electrode consistently maintained clear and symmetric redox peaks without significant distortion, demonstrating that the material operates via a typical Faradaic pseudocapacitive energy storage mechanism. Furthermore, as the scan rate increased, the peak current increased synchronously while the peak positions showed only slight shifts, indicating that the NiCoS_x_/NF electrode possesses excellent reaction reversibility and ultrafast ion/electron transfer kinetics. Figure 4b compares the CV curves of five electrodes-NiCo_3_S_x_/NF, NiCo_2_S_x_/NF, NiCoS_x_/NF, Ni_2_CoS_x_/NF, and Ni_3_CoS_x_/NF-at a scan rate of 10 mV s^−1^. All samples exhibited prominent redox peaks, highlighting the pseudocapacitive behavior characteristic of cobalt sulfide nanosheet electrodes. These redox peaks are attributed to the reversible oxidation-reduction reactions between Ni^2+^/Ni^3+^ and Co^2+^/Co^3+^. Among them, the NiCoS_x_/NF sample displayed the largest CV integral area and the highest peak current, proving that this optimal Ni/Co ratio provides the greatest number of electrochemically active sites and the strongest charge storage capability, reflecting the most prominent synergistic optimization effect. The CV curves indicate that NiCoS_x_/NF possesses superior electrochemical activity and storage capacity. This exceptional performance stems from its stable nanosheet structure, which ensures full exposure of active sites while promoting rapid ion and electron transport, and maintains structural integrity during repeated redox reactions, preventing the detachment of active sites or structural collapse. As shown in Figure 4c, at current densities of 0.5–10 A g^−1^, the NiCoS_x_/NF electrode shows highly symmetric nonlinear charge–discharge plateaus, which align well with the pseudocapacitive behavior revealed by CV results. Even at a high current density of 10 A g^−1^, the electrode retains a complete charge discharge profile with low polarization, intuitively demonstrating its excellent rate tolerance and structural stability. Figure 4d shows that under the same test conditions at 0.5 A g^−1^, the NiCoS_x_/NF electrode delivers the longest discharge duration and the highest specific capacity among the tested samples, significantly outperforming other modified samples with varying Ni/Co ratios. The good symmetry and small voltage drop of the charge discharge curves for all samples further verify that a moderate Ni/Co atomic ratio can substantially reduce the internal resistance of the electrode, enabling a reversible and efficient redox energy storage reaction [36]. In summary, the two-dimensional nanosheet-like NiCoS_x_/NF electrode demonstrates optimal energy storage performance due to its optimized electronic structure, abundant reaction sites, and open ion transport channels. Rational modulation of the Ni/Co molar ratio is key to further amplifying the bimetallic synergistic effect and simultaneously enhancing the specific capacity and rate performance of the electrode [37]. Its nanosheet structure can effectively mitigate volume changes during charge discharge processes, prevent structural damage, and ensure unobstructed ion transport pathways, thereby achieving efficient and stable charge discharge cycling (Figure S6).

**Figure 5:**
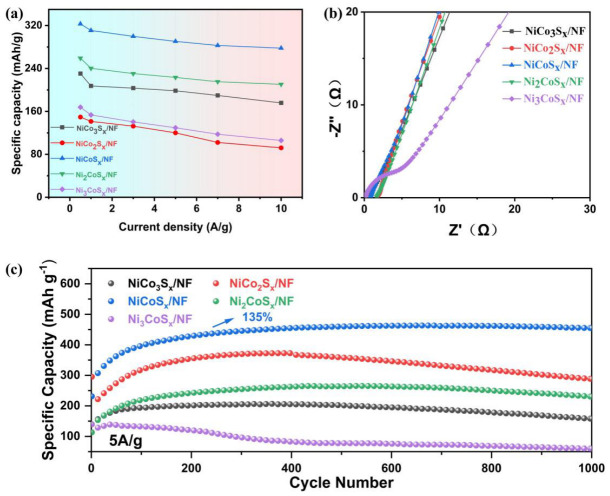
Electrodes with different Ni/Co ratios: (**a**) rate performance; (**b**) Nyquist plots; (**c**) cycling performance.

The rate capability of electrodes with different Ni/Co molar ratios was tested in a 6 M KOH electrolyte, and the results are presented in Figure 5a. A significant electrochemical discrepancy was observed for electrodes with different Ni/Co molar ratios at current densities of 0.5, 1, 3, 5, 7 and 10 A g^−1^. The Ni_2_CoS_x_/NF electrode delivers an initial specific capacity of 230.39 mAh g^−1^, which gradually decreases to 175.83 mAh g^−1^ at 10 A g^−1^, with a capacity retention of 76.32%. For the Ni_3_CoS_x_/NF electrode, the specific capacity declines from 149.53 mAh g^−1^ to 92.08 mAh g^−1^ at 10 A g^−1^, corresponding to a capacity retention of 61.58%. The NiCo_2_S_x_/NF electrode possesses desirable rate performance, with the specific capacity reducing from 259.22 mAh g^−1^ to 210.42 mAh g^−1^ and a high capacity retention of 81.17%. As for NiCo_3_S_x_/NF, its specific capacity fades from 167.78 mAh g^−1^ to 105.83 mAh g^−1^, achieving a capacity retention of 63.07%. Notably, the NiCoS_x_/NF electrode presents remarkably outstanding electrochemical performance under the same test conditions. It delivers a high specific capacity of 323.02 mAh g^−1^ at 0.5 A g^−1^, and still maintains a high capacity output of 277.92 mAh g^−1^ even when the current density increases to 10 A g^−1^. This outstanding rate capability is attributed to its stable nanosheet structure, which maintains structural integrity even at high current densities, preventing structural damage caused by accelerated ion transport rates and ensuring stable capacity output across various rates, highlighting the immense potential of this stable sheet-like architecture for practical applications. Electrochemical impedance spectroscopy (EIS) of the electrodes was investigated over a frequency range of 0.01 Hz to 100 kHz under open-circuit voltage conditions, with the results presented in Figure 5b. In Nyquist plots, the diameter of the high-frequency semicircle represents the charge transfer resistance (R_ct_), the equivalent series resistance (R_s_) is represented by the intersection of the semicircle with the real axis, and the sloped line represents the diffusion resistance, with its slope being inversely proportional to the diffusion resistance. All electrode samples exhibited low Rs values, benefiting from the in situ growth of the samples on the nickel foam substrate, which effectively reduced the electrode resistance. The absence of distinct semicircles in the curves of all samples indicates a rapid charge transfer process and fast electrode kinetics, with significantly reduced Rct. Fitting analysis performed using ZView software (ZView v.40h) shows that the NiCoSx/NF electrode has an Rct value of only 0.43 Ω in Figure S5, and it possesses the largest slope, indicating minimal diffusion resistance and optimal conductivity. This result is closely related to the stable nanosheet structure [38]: the intact and interconnected sheet-like architecture constructs efficient electron and ion transport channels, while its structural stability prevents these channels from being blocked due to structural damage, thereby significantly reducing both charge transfer and diffusion resistances. Cycling performance tests were conducted on the products using a three-electrode system. Figure 5c displays the cycling performance of five electrodes with different Ni/Co ratios after 50 activation cycles at 5 A g^−1^. In the initial stage, the capacities were as follows: NiCo_3_S_x_/NF at 140 mAh g^−1^, NiCo_2_S_x_/NF at 295 mAh g^−1^, NiCoS_x_/NF at 231 mAh g^−1^, Ni_2_CoS_x_/NF at 113 mAh g^−1^, and Ni_3_CoS_x_/NF at 139 mAh g^−1^, with NiCo_2_S_x_/NF showing the highest initial capacity [39]. After 100 cycles, the NiCoS_x_/NF capacity retention rate is 135% of the initial capacity, indicating stable performance; before 100 cycles, the electrode gradually activated during repeated charge discharge processes, leading to a significant increase in capacity. In contrast, the Ni_3_CoS_x_/NF electrode experienced a sharp decline after a brief capacity increase around 200 cycles. The capacity trends of NiCo_2_S_x_/NF and NiCoS_x_/NF differed, and the capacity of NiCo_2_S_x_/NF was slightly lower than that of NiCoS_x_/NF. After 50 cycles of activation, which fully activate the electrode material, the gradual increase in capacity is due to the electrolyte continuously penetrating the internal channels of the two-dimensional nanosheets, fully exposing the active sites, making the ion transport channels increasingly smooth, thereby improving the utilization of active materials. At the same time, the vertically aligned nanosheet structure gradually adapts to the repeated insertion and extraction of ions, optimizing the reaction kinetics. The excellent cycling stability of the NiCoS_x_/NF electrode primarily stems from its stable nanosheet structure, which can effectively withstand mechanical stress during charge discharge cycles, preventing structural collapse or detachment of active materials, thus ensuring high capacity retention after long-term cycling [40]. After assembling the five types of electrodes with different Ni/Co ratios into alkaline zinc-based batteries, the experimental results showed that NiCoS_x_/NF maintained outstanding capacity and rate performance in the alkaline battery system, exhibiting the best overall performance [38]. This fully demonstrates the broad application prospects of this stable sheet-like structure in the field of alkaline batteries.

### 3.3. Full Battery Performance Testing and Analysis

**Figure 6:**
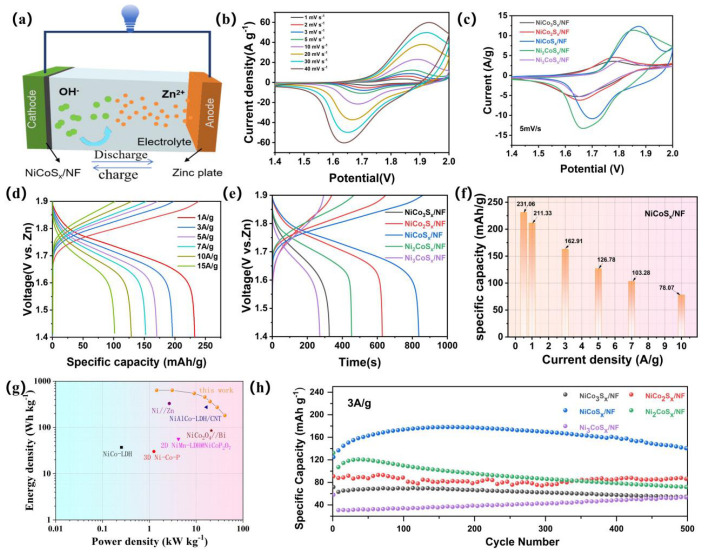
(**a**) Schematic diagram of the NiCoS_x_ battery; (**b**) CV curves of the NiCoS_x_/NF//Zn battery; (**c**) comparison of CV curves for different Ni/Co ratios; (**d**) GCD curves of the NiCoS_x_/NF//Zn battery; (**e**) GCD curves for different Ni/Co ratios; (**f**) rate performance curves for different Ni/Co ratios; (**g**) Ragone plot of the NiCoS_x_/NF//Zn battery and comparison with other works; (**h**) cycle performance of batteries with different Ni/Co ratios.

To investigate the practical application performance of NiCoS_x_/NF in real devices, the electrode films were cut into small discs with an area of 1.13 cm^2^ and placed at the cathode position of a coin cell. A drop of electrolyte was added to ensure full wetting, followed by placing a hydrophilic separator which was also wetted with a drop of electrolyte. The zinc anode disc was then positioned on top of the separator at the anode side of the coin cell, and the assembly was sealed using a crimping machine. Using a two-electrode system, with NiCoS_x_/NF as the positive electrode and commercial zinc foil as the negative electrode, and employing 4 M KOH+Sat. Zinc Acetate as the electrolyte, alkaline zinc-based batteries were assembled, as shown in Figure 6a. Under identical electrolyte and zinc anode conditions, five electrodes with different Ni/Co ratios were subjected to comparative CV (5 mV s^−1^) and GCD (1 A g^−1^) tests. Figure 6b shows CV curves of the NiCoS_x_/NF//Zn cell at 1–40 mV s^−1^ within 1.4–2.0 V. At a scan rate of 1 mV s^−1^, the redox peaks exhibit good symmetry, indicating balanced electrochemical reactions and a stable process. When the scan rate is increased to 40 mV s^−1^, the redox peaks remain symmetric, demonstrating the battery’s excellent reversibility and its ability to maintain stable electrochemical reactions under rapid potential changes. Moreover, the CV profile shape is well maintained across scan rates of 1–40 mV s^−1^, demonstrating excellent electrochemical stability of the battery. This stability originates from the stable nanosheet structure of the positive NiCoS_x_/NF, which maintains structural integrity under potential variations corresponding to different scan rates, ensuring consistency in electrochemical reactions. Figure 6c compares the full-cell CV curves at a scan rate of 5 mV s^−1^ within the operating potential range of 1.4–2.0 V. The CV integral areas for the NiCo_2_S_x_/NF and NiCoS_x_/NF electrodes are significantly larger than those of other electrodes, indicating higher electrochemical activity and a greater quantity of participating active materials. Figure 6d presents the charge discharge curves of the NiCoS_x_/NF//Zn battery at various current densities. The battery exhibits excellent charge discharge efficiency, further confirming the superior charge storage capability of the NiCoS_x_/NF electrode and highlighting the critical role of the nanosheet structure in enhancing specific capacity and cycling stability [41]. The high Coulombic efficiency indicates efficient ion diffusion and charge transfer capabilities, all of which are underpinned by the stable sheet-like structure that ensures unobstructed transport channels and structural integrity (Figure S7). In Figure 6e, the discharge time of NiCoS_x_/NF//Zn is noticeably longer than that of batteries with other ratios approximately twice that of the Ni_2_CoS_x_/NF//Zn battery while the NiCo_3_S_x_/NF//Zn and Ni_3_CoS_x_/NF//Zn batteries show shorter discharge times and lower capacities [42]. The superior performance of the NiCoS_x_/NF electrode in the full battery once again underscores the pivotal role of the stable nanosheet structure: in a practical battery system, this structure maintains long-term stability, preventing structural degradation due to electrolyte erosion or charge discharge cycling, thereby ensuring high activity and high capacity output. The rate performance of the NiCoS_x_/NF//Zn battery was tested, with results shown in Figure 6f. At current densities of 0.5, 1, 3, 5, 7, and 10 A g^−1^, the battery delivered high capacities of 231.06, 211.33, 162.91, 126.78, 103.28, and 78 mAh g^−1^, respectively. These results align closely with the previous battery performance conclusions, further validating the exceptional performance of the NiCoS_x_/NF electrode under varying conditions, with the stable nanosheet structure serving as the core guarantee for maintaining high capacity output at different rates [43]. To further highlight the exceptional characteristics of the NiCoS_x_/NF//Zn battery in aqueous electrochemical systems, we conducted an in-depth analysis using a Ragone plot. As clearly seen in Figure 6g, based on calculations using the mass of the NiCoS_x_/NF//Zn cathode, the battery investigated in this work demonstrates remarkable performance metrics. It achieves a maximum energy density of 615.2 Wh kg^−1^ and a maximum power density as high as 38.6 kW kg^−1^ (based on the mass of positive electrode active materials). Comparing these data horizontally with other reported batteries reveals a distinct advantage: for instance, an alkaline Ni-Zn battery delivers an energy density of 303.8 Wh kg^−1^ at a power density of 2.69 kW kg^−1^ [44]; a 3D Ni-Co-P battery offers only 30.2 Wh kg^−1^ at 1.262 kW kg^−1^ [45]; and a NiCo-LDH battery provides 37.02 Wh kg^−1^ at 0.256 kW kg^−1^ [46]. In contrast, the performance indicators of the NiCoS_x_/NF//Zn battery almost comprehensively surpass those of these previously reported types [47]. This fully demonstrates the prominent advantages of the NiCoS_x_/NF//Zn battery within aqueous electrochemical systems. A 500-cycle test was performed on the NiCoS_x_/NF//Zn battery at a low current density of 3 A g^−1^, with results shown in Figure 6h [48,49,50]. Compared to batteries assembled with the other four electrodes, the NiCoS_x_/NF//Zn battery exhibits a significant advantage in cycling performance. In the initial stage, the battery capacity reached a high level of 125 mAh g^−1^, and after 500 cycles, the capacity increased to 140 mAh g^−1^, resulting in a capacity retention of 112.5% [51,52,53]. Due to the presence of numerous interlayer voids in the vertically open two-dimensional hierarchical nanosheet array, the alkaline electrolyte continuously penetrates the material during repeated charge discharge cycles, and originally inert active sites are continuously electrochemically activated. At the same time, the ultrathin flexible nanosheets can effectively buffer the volume changes caused by zinc ion intercalation and deintercalation, allowing the electrode structure to remain intact, thereby resulting in a capacity retention of 112.5% [54,55,56]. This exceptional cycling performance fully proves that the stable nanosheet structure of NiCoS_x_/NF can maintain structural integrity during long-term charge discharge cycling, effectively mitigating volume changes and active material loss . It may even undergo further activation during the cycling process, leading to capacity growth, thus highlighting the decisive role of the stable sheet-like structure on the battery’s cycling performance.

## 4. Conclusions

In this work, an innovative strategy combining solvothermal synthesis with in situ hydrolysis was employed to successfully achieve the in situ growth of nanosheet-like cobalt sulfide electrodes on a nickel foam substrate. The two-dimensional nanosheet arrays significantly increase the exposure of electrochemically active sites, while the bimetallic Ni-Co electronic synergistic coupling optimizes charge transfer kinetics. The stable lattice framework constructed via in situ topotactic sulfuration effectively mitigates volume deformation and structural pulverization during charge discharge processes. At a current density of 0.5 A g^−1^, the NiCoS_x_/NF electrode delivers a high specific capacity of 323 mAh g^−1^. After undergoing an activation process of 50 cycles, when the current density is increased to 5 A g^−1^, the electrode’s capacity still reaches 231 mAh g^−1^, and after 100 cycles, its capacity retention remains as high as 135%. When assembled into a full cell, the NiCoS_x_/NF//Zn battery exhibits a high specific capacity of 231 mAh g^−1^ at 0.5 A g^−1^. Even when the current density is increased to 3 A g^−1^, the battery maintains an exceptionally high specific capacity of 125 mAh g^−1^. After 500 cycles, the NiCoS_x_/NF//Zn battery demonstrates a capacity retention of 112.5%. This two-dimensional sheet-like structure combined with in situ topotactic sulfuration is key to enhancing the reaction kinetics and cycling stability of cobalt sulfide cathodes, providing a concise and scalable synthetic approach and mechanistic support for designing high-capacity, long-life, and high-rate alkaline zinc battery electrodes.

## Data Availability

The original contributions presented in this study are included in the article/Supplementary Materials. Further inquiries can be directed to the corresponding authors.

## Supplementary Materials

The following supporting information can be downloaded at: https://www.mdpi.com/article/10.3390/nano16120766/s1, Figure S1. SEM images (a) NiCo/NFprecursor (b) NiCo_3_S_x_/NF (c) NiCo_2_S_x_/NF (d) NiCoS_x_/NF (e) Ni_2_CoS_x_/NF (f) Ni_3_CoS_x_/NF. Figure S2. (a) SEM image and (b) XPS survey spectrum of the NiCoS_x_/NF sample. Figure S3. CV curves of electrodes with different Ni/Co ratios (a) NiCo_3_S_x_/NF (b) NiCo_2_S_x_/NF (c) NiCoS_x_/NF (d) Ni_2_CoS_x_/NF (e) Ni_3_CoS_x_/NF (f) CV curves at a scan rate of 10 mV s^−1^. Figure S4. GCD curves of electrodes with different Ni/Co ratios (a) NiCo_3_S_x_/NF (b) NiCo_2_S_x_/NF (c) NiCoS_x_/NF; (d) Ni_2_CoS_x_/NF (e) Ni_3_CoS_x_/NF (f) GCD curves at a current density of 0.5 A g^−1^. Figure S5. Fitted Nyquist plot. Figure S6. SEM image of the NiCoS_x_/NF electrode after 1000 cycles. Figure S7. EIS plot of the NiCoS_x_/NF electrode after 1000 cycles. Table S1. Comparison of the electrochemical performance of single-metal sulfides. Refs. [48,49,50,51,52,53,54,55,56] are cited in the supplementary materials.
