# Supplementary material for: Construction of Vertical 2D Open Hierarchical NiCoSx Nanosheet Arrays for High-Performance Alkaline Zinc Batteries
Source: Nanomaterials (Basel). 2026 Jun 18;16(12):766. doi: 10.3390/nano16120766 (PMC13305964; doi:10.3390/nano16120766)
Supplement: Supplementary file 1 [file nanomaterials-16-00766-s001.zip › nanomaterials-4322268-supplementary.pdf]

Supplementary Materials

Construction of Vertical 2D Open Hierarchical $NiCoS_x$ Nanosheet Arrays for High-Performance Alkaline Zinc Batteries

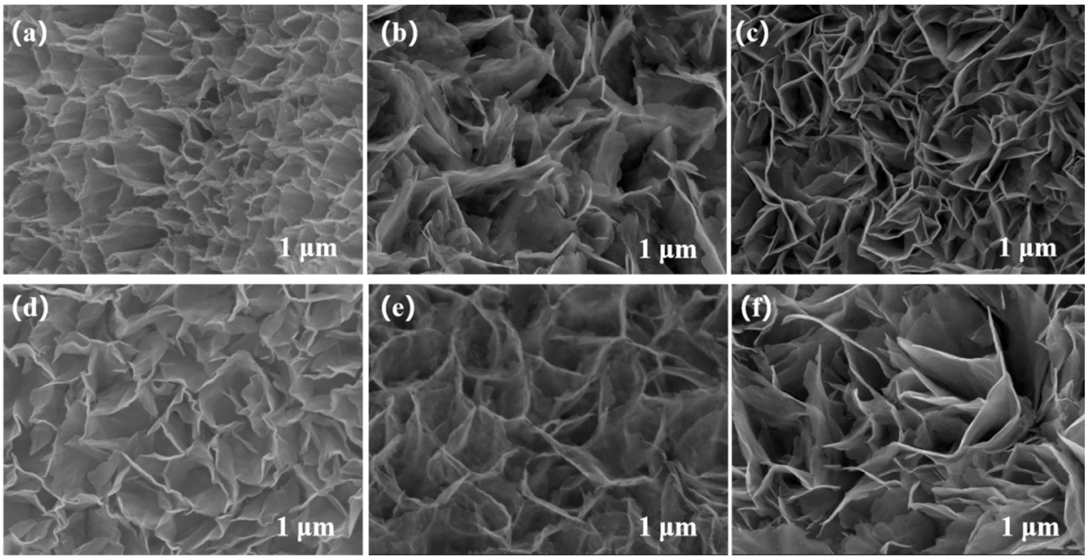

Figure S1. SEM images (a) NiCo/NFprecursor; (b) $NiCo_3S_x$/NF; (c) $NiCo_2S_x$/NF; (d) $NiCoS_x$/NF; (**e**) Ni_2CoS_x/NF; (f) Ni_3CoS_x/NF.

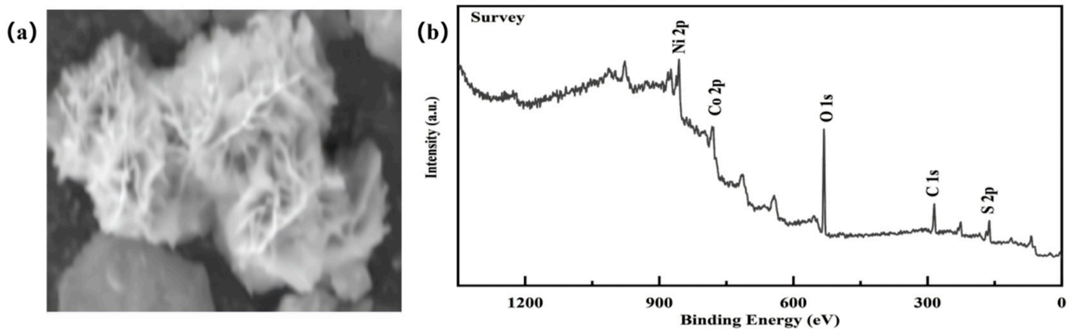

Figure S2. (a) SEM image and (b) XPS survey spectrum of the $NiCoS_x$/NF sample.

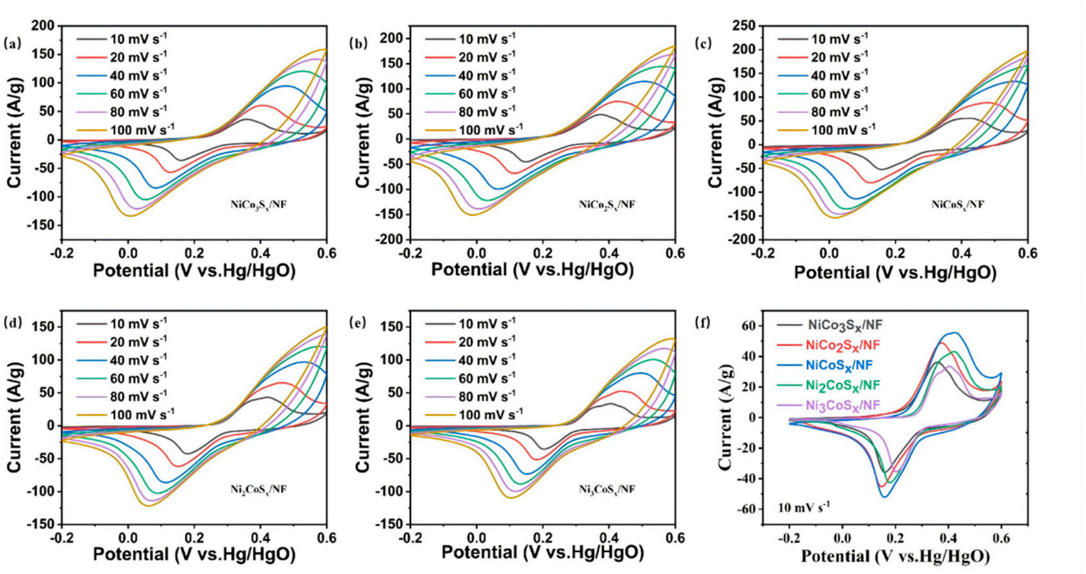

Figure S3. CV curves of electrodes with different Ni/Co ratios:

(a) $NiCo_3S_x$/NF; (b) $NiCo_2S_x$/NF ;(c) $NiCoS_x$/NF; (d) Ni_2CoS_x/NF; (e) Ni_3CoS_x/NF; (f) CV curves at a scan rate of 10 mV s^{-1}.

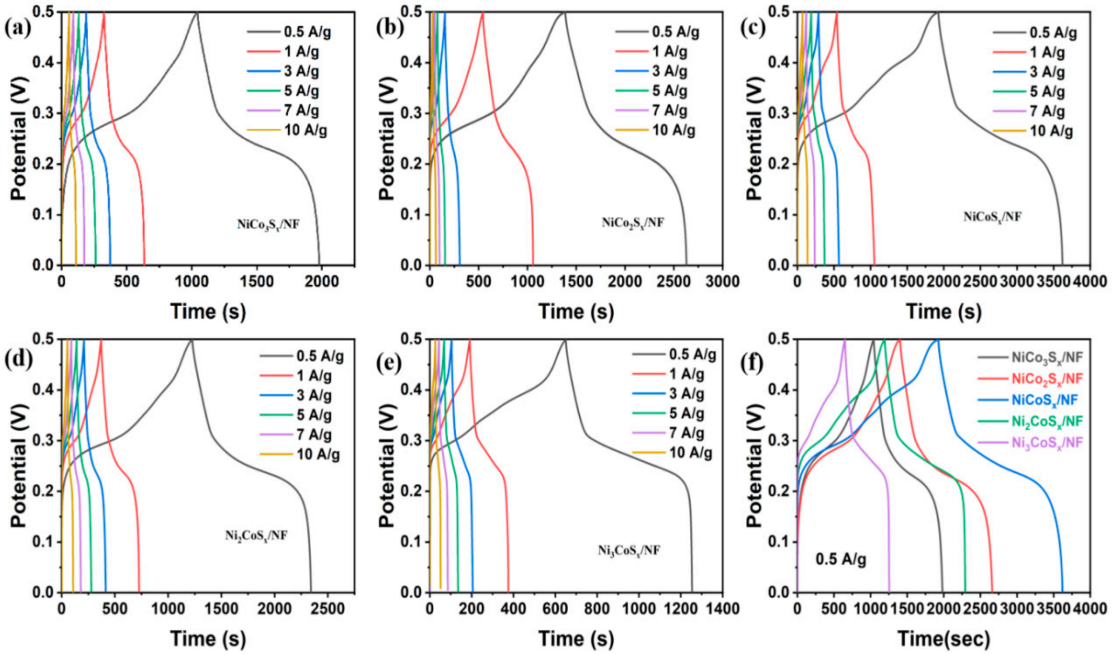

Figure S4. GCD curves of electrodes with different Ni/Co ratios:

(a) $NiCo_3S_x$/NF; (b) $NiCo_2S_x$/NF; (c) $NiCoS_x$/NF; (d) Ni_2CoS_x/NF; (e) Ni_3CoS_x/NF; (f) GCD curves at a current density of 0.5 A g^{-1}.

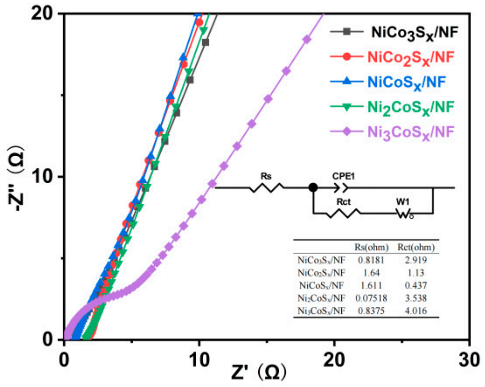

Figure S5. Fitted Nyquist plot.

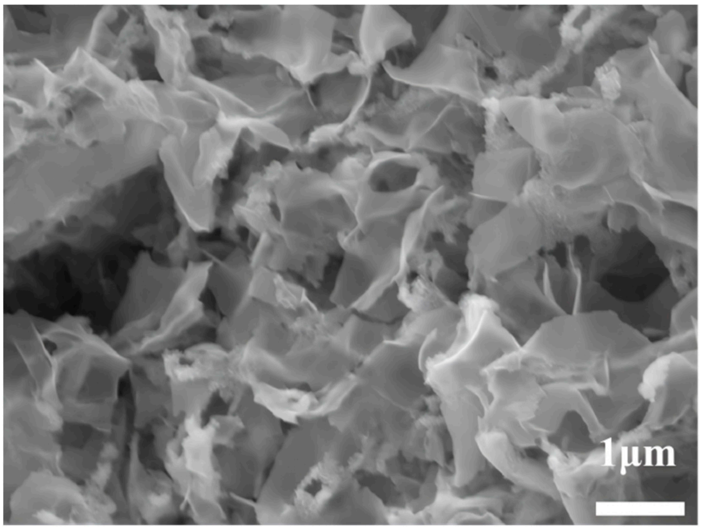

Figure S6. SEM image of the $NiCoS_x$/NF electrode after 1000 cycles.

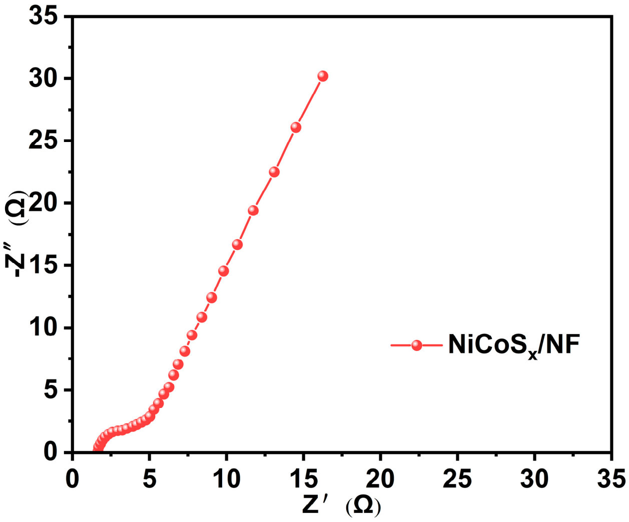

Figure S7. EIS plot of the $NiCoS_x$/NF electrode after 1000 cycles.

Table. S1. Comparison of the electrochemical performance of single-metal sulfides.

material	Cycle stability	Reference
**$NiCoS_x/NF$**	**112.5% capacity retention at 3 A g^{-1} after 500 cycles**	**This work**
NiS-coated $Ni_{0.95}Zn_{0.05}(OH)_2$//Zn mesh	97.8% capacitance retention for 500 cycles with 1 mA cm^{-2}	[1]
Co_3S_4//Zn foil	≈96% capacity retention at 1 A g^{-1} after 500 cycles	[2]
Co-Ni_3Se_2//Zn foil	77.9% capacity retention at 3 A g^{-1} after 100 cycles	[3]
NiS	≈66.8% capacity retention at 10 A g^{-1} after 500 cycles	[4]
Mo_2S_3@Ni_3S_2 nanowire	90% capacity retention at 1 A g^{-1} after 500 cycles	[5]
Nest-like hierarchical Ni_3S_2	78% capacity retention for 500 cycles with 25 mA cm^{-2}	[6]
Clustered network-like Ni_3S_2–Co_9S_8	96% capacity retention for 500 cycles with 5 mA cm^{-2}	[7]
Hierarchical Co_3O_4@Ni_3S_2 core/shell nanowire arrays	92% capacity retention at 4 A g^{-1} after 500 cycles	[8]
$NiCo_2S_4$//G/CS ASC	95% capacity retention at4 A g^{-1} after 500 cycles	[9]

References

[1] Mao, J., Iocozzia, J., Huang, J., Meng, K., Lai, Y., & Lin, Z. (2018). Graphene aerogels for efficient energy storage and conversion. Energy & Environmental Science, 11(4), 772-799. https://doi.org/10.1039/C7EE03031B.

[2] Zhang, S. W., Yin, B. S., Luo, Y. Z., Shen, L., Tang, B. S., Kou, Z., Gong, H.; et al. (2020). Fabrication and theoretical investigation of cobaltosic sulfide nanosheets for flexible aqueous Zn/Co batteries. Nano Energy, 68, 104314.https://doi.org/10.1016/j.nanoen.2019.104314.

[3] Miao, Y., Zhang, X., Zhan, J., Sui, Y., Qi, J., Wei, F., Sun, Z.; et al. (2020). Hierarchical NiS@ CoS with controllable core-shell structure by two-step strategy for supercapacitor electrodes. Advanced Materials Interfaces, 7(3), 1901618.https://doi.org/10.1002/admi.201901618.

[4] Pothu, R., Bolagam, R., Wang, Q. H., Ni, W., Cai, J. F., Peng, X. X., Ma, J.; et al. M. (2021). Nickel sulfide-based energy storage materials for high-performance electrochemical capacitors: R. Pothu et al. Rare Metals, 40(2), 353-373.https://doi.org/10.1007/s12598-020-01470-w.49.

[5] NNiu, S. F., & Zheng, J. H. (2018). Mo_2S_3@Ni_3S_2 nanowries on nickel foam as a highly-stable supercapacitor material. Journal of Alloys and Compounds, 737, 809-814.https://doi.org/10.1016/j.jallcom.2017.12.051.

[6] Krishnamoorthy, K., Veerasubramani, G. K., Radhakrishnan, S., & Kim, S. J. (2014). One pot hydrothermal growth of hierarchical nanostructured Ni_3S_2 on Ni foam for supercapacitor application. Chemical Engineering Journal, 251, 116-122.https://doi.org/10.1016/j.cej.2014.04.006.

[7] Han, T., Jiang, L., Jiu, H., & Chang, J. (2017). Hydrothermal synthesis of the clustered network-like Ni_3S_2-Co_9S_8 with enhanced electrochemical behavior for supercapacitor electrode. Journal of Physics and Chemistry of Solids, 110, 1-8.https://doi.org/10.1016/j.jpcs.2017.05.024

[8] Zhang, J., Lin, J., Wu, J., Xu, R., Lai, M., Gong, C., Zhou, P.; et al. (2016). Excellent electrochemical performance hierarchical Co_3O_4@Ni_3S_2 core/shell nanowire arrays for asymmetric supercapacitors. Electrochimica Acta, 207, 87-96.https://doi.org/10.1016/j.electacta.2016.04.068.

[9] 56.Shen, L., Yu, L., Wu, H. B., Yu, X. Y., Zhang, X., & Lou, X. W. (2015). Formation of nickel cobalt sulfide ball-in-ball hollow spheres with enhanced electrochemical pseudocapacitive properties. Nature communications, 6(1), 6694.DOI: 10.1038/ncomms7694
